# Reconstruction of the Passive Layer of AISI 304 and 316 Steel After Scratching

**DOI:** 10.3390/ma17246238

**Published:** 2024-12-20

**Authors:** Sylwia Charazińska, Andrzej Sikora, Beata Malczewska, Paweł Lochyński

**Affiliations:** 1 Institute of Environmental Engineering, Wrocław University of Environmental and Life Sciences, pl. Grunwaldzki 24, 50-363 Wroclaw, Poland; sylwia.charazinska@upwr.edu.pl (S.C.); beata.malczewska@upwr.edu.pl (B.M.); 2 Department of Nanometrology, Faculty of Electronics, Photonics and Microsystems, Wrocław University of Science and Technology, 50-370 Wrocław, Poland; andrzej.sikora@pwr.edu.pl

**Keywords:** stainless steel, electropolishing, XPS, depth profile, AFM, high-resolution spectra, nanoscratching, passive film

## Abstract

Austenitic stainless steels are used widely in many fields due to their good mechanical properties and high resistance to corrosion. This work focuses on the reconstruction of the passive film after scratching. The purpose of the study was to compare changes in the rate of passive layer reconstruction and to discuss the effect of both the type of material and its electrochemical treatment on the reconstruction of the passive layer for two types of stainless steel: 304 and 316. The XPS tests performed indicate a significantly higher Cr/Fe ratio for the samples after the electropolishing process of 1.41–1.88 compared to the as-received samples of 0.82–0.86. After 2–3 min of sputtering the surface with Ar+ ions, a decrease in chromium content can be observed, with a simultaneous increase in nickel content, visible especially for the electropolished samples. A new approach in the conducted research was to scratch the test samples under controlled conditions, then evaluate the dynamics of the passive layer reconstruction using the AFM method, and then confront the obtained results with XPS measurements for the corresponding samples. For the as-received samples (2B finish) and those after surface treatment, regardless of the level of contamination of the electropolishing process bath, the reconstruction time was similar, which was approximately 2 h, although certain differences in the process dynamics were noticeable.

## 1. Introduction

Stainless steel (SS) is ubiquitous in today’s industrial world, as it is generally considered to be corrosion-resistant. This is a result of the relationship between the iron alloy and environmental factors. The corrosion resistance of SS results from the ‘passive’ chromium-rich oxide layer that forms naturally on the surface of the steel. After its initial formation, its thickness increases over time, which is what its mechanical properties depend on [1,2,3]. Other properties of the passive layer, such as adherence and stability, determine the SS’s level of corrosion resistance [4].

Despite numerous studies, the mechanism of passive layer formation has not been sufficiently understood [2]. In a recent study, Jamebozorgi et al. [5] suggested that the underlying mechanisms of passive layer formation are based on clustering and local detachment of metallic atoms. In contrast, Suresh et al. [6] demonstrated that the formation and breakdown of a passive film layer are mainly controlled by ionic and electronic transport reactions [1,6]. Numerous studies have described the passive film on SS as a bilayer structure [5,7,8,9,10,11,12,13]. Passive layers typically exhibit a thickness of a few to several micrometers, are non-porous, show good tightness and chemical resistance, and have good electron conductivity [14]. According to a recent study by Rahimi et al. [15], the cross-section of the passive layer can be separated into an n-type outer layer and a p-type inner layer, based on their chemical composition.

Under typical atmospheric conditions, the passive layer can regenerate when oxygen is present in air. It is produced when oxygen in the air and chromium in the SS undergo a chemical reaction. Chromium oxide is a product of these reactions. Consequently, the inner oxide, which is rich in chromium, is recognized as a corrosion barrier. The fundamental mechanisms in passive layer formation require a more thorough theoretical examination. This is directly related to the limitations of analytical and spectroscopic approaches in investigating nanoscale kinetic processes [5]. Numerous studies have investigated the kinetics of SS oxidation. For example, a recent study by Tcharkhtchi-Gillard et al. [16] concluded that at higher potentials, transpassive behavior was denoted, characterized by a high mass-loss rate and a specific intergranular corrosion morphology. They performed chronoamperometry experiments coupled with mass loss measurements to quantify the oxidation and reduction kinetics. On the other hand, Kim et al. [17] evaluated the growth kinetics of passive films on iron and stainless steel using in situ specular X-ray reflectivity. They reported that in SS, the oxide properties changed as the applied potential varied. Furthermore, Jaffré et al. [18] showed that the electrochemical reactivity of the grown oxide is affected by the chemistry of the environment more than by the mechanical surface treatment. As discussed by Wang et al. [19], oxygen pressure and temperature have the most impact on the oxidation rate.

When a passive layer of 304 or 316 stainless steel is scratched, it can regrow. This process may be hampered if the passive layer is exposed to high temperatures and chloride ions. For instance, the protective capacity of these passive films may be further diminished by a combination of halide ions and surface mechanical damage caused by friction/scratching [1,20]. Cl^−^ can easily penetrate the passive film and then combine with cations in the passive film to form soluble chloride, breaking the integrity of the passive film, and thus causing pitting corrosion to occur [21]. Additionally, factors influencing the susceptibility of steel to the local failure of the passive layer comprise non-metallic inclusions, the presence of second-phase particles, and structural inhomogeneity [22]. Moreover, Domańska et al. [23] indicated that the observed pitting corrosion of stainless steel samples placed over the surface of municipal wastewater was a consequence of having been in an environment containing hydrogen sulfide. The passive layer can be strengthened by the passivation process, that is, chemical or electrochemical protection of the steel surface [24].

The electropolishing process can be used to remove micro-scratches and clean the metallic surface of stainless steel, followed by the formation of a new protective layer with improved properties. The principles of electrolysis govern the process, which necessitates current density and the use of an electrolyte, usually consisting of sulfuric and orthophosphoric acids. A layer of metal is removed from a workpiece’s surface by electropolishing, while an electric current is applied [25]. Previous research has highlighted the importance of electrochemical polishing process parameters [25,26,27,28,29].

As previously reported in the literature, different practices have been applied to prevent the corrosion of metals. Among them, the applications of protective coatings, electroplating, anodizing, control of environmental conditions, and use of anti-corrosion systems or metal alloys are the most common [21,30,31,32,33,34,35]. Pantoja et al. [36] reported that chemical pretreatments increase the surface energy, primarily because of the removal of most external layers, which causes the passive layer to be strongly Cr-rich. The steel’s strength, hardness, hardenability, and ductility are all improved by the addition of nickel, which also lessens the steel’s brittleness. Steel that contains molybdenum is more resistant to high temperatures, hydrogen sulfide, and embrittlement. Therefore, the molybdenum content is the primary manner of distinguishing between 304 and 316 grade stainless steel. Other additives often used are silicon, manganese, niobium, nitrogen, sulfur, and phosphorus. Moslehifard et al. [37] found that Co-Cr-Mo alloys exhibit better corrosion resistance than Ni-Cr-based alloys. Wang et al. [38] reported that the addition of nitrogen results in the replacement of nickel which, in turn, causes strong stabilization of the austenitic microstructure. When Xia et al. [39] investigated the effect of adding a small amount of nickel during sintering, they discovered an improvement in corrosion resistance, especially against acids. For the same environment, the addition of molybdenum and tungsten further improved resistance to corrosion. Pardo [40] reported that the addition of molybdenum increased the corrosion resistance of stainless steels in acidic medium, unlike the addition of manganese. On the other hand, Robin et al. [41], when examining the impact of the silicon presence in steel, revealed the presence of silicon in the passive layer, which decreased the chromium content. Additionally, they found that high Si-containing austenitic stainless steels had good corrosion resistance, which may have been caused by a drop in the cathodic reaction rate. In comparison to uncoated specimens, Padhy et al. [9] have reported an increase in the corrosion current density of sputter-deposited TiO_2_ on austenitic type 304 L SS in a nitric acid medium.

To assess the chemical composition and thickness of the oxide layer on SS, different methods may be applied, but the Cr/Fe atomic ratio, Cr oxide/Fe oxide ratio, and total oxide thickness are highly relevant indicators. Depending on the emitted signal, the following methods are distinguishable: X-ray photoelectron spectroscopy (XPS—X-ray photoelectron spectroscopy), Auger electron spectroscopy (AES—Auger electron spectroscopy), ultraviolet excited electron spectroscopy (UPS—Ultraviolet Photoelectron Spectroscopy), secondary ion mass spectroscopy (SIMS), ion scattering spectroscopy (ISS), and sputtered neutral particle mass spectroscopy (SNMS) [13,42,43]. The film thickness of the passive layer is a crucial factor. Therefore, the use of appropriate measurement techniques is essential. Yao et al. [44] have proposed a real-time evolution and characterization of passive films, in which they applied magnetic force microscopy, Kelvin probe force microscopy, SEM, EDS, and AES. Studying this phenomenon, they found that changes over time in the passive layer can be divided into three periods, the first nucleation, the second rapid growth, and the third stable growth. In recent years, the most commonly used method for evaluating the passive layer has been XPS [13,38,42]. Information about the depth profile and surface is provided by XPS. It has an analysis depth of roughly 2 to 4 nm and measures the energy spectra of electrons released when the surface is exposed to X-rays. As it is the second most popular technique, AFM offers numerous opportunities to investigate and evaluate the material structure. It is employed to study passive films at the metal/environment interface that are of nanometer order [19,45,46,47,48].

Despite the numerous related studies, the issue of the SS reconstruction rate has not received enough attention. The present study’s objective was to compare the dynamics of the reconstruction changes with changes in the passive layer reconstruction rate for 304 and 316 steels, including the 2B finish, following electropolishing. With the use of XPS measurements to describe the film’s structure, the kind of material and its electrochemical treatment on the passive layer reconstruction have been further examined in this work. Furthermore, samples were scratched under controlled settings in the current study, before the kinetics of the passive layer reconstruction were assessed.

## 2. Materials and Methods

### 2.1. Materials

Samples were obtained from cold-rolled 1.5 mm thick AISI 304 (Aperam Stainless France, Isbergues, France) and AISI 316 (Aperam, Genk, Belgium) stainless steel with a 2B surface finish. The chemical material composition of 304 stainless steel was the following (wt%): 18.23% Cr, 8.02% Ni, 1.39% Mn, 0.37% Si, 0.201% Co, 0.066% N, 0.034% P, 0.028% C, 0.002% S, and balance Fe. The composition of 316 steel was, respectively, the following (wt%): 16.57% Cr, 10.04% Ni, 2.03% Mo, 1.32% Mn, 0.032% N, 0.48% Si, 0.030% P, 0.018% C, 0.003% S, and balance Fe.

### 2.2. Sample Preparation

Tests were conducted on round samples with a diameter of 16.7 mm, with a hole of 1 mm diameter located 1 mm away from the edge of the sample. 304 and 316 stainless steel, as-received samples, were degreased with acetone and then rinsed in distilled water for 20 min in an ultrasonic bath. The acetone rinsing and degreasing procedure was also carried out for the samples prior to the electropolishing process. The process bath for electropolishing contained sulfuric acid and phosphoric acid. The electropolishing process parameters were as follows: temperature 55 °C, process time 15 min, current density 8 A/dm^2^. The electropolishing circuit consisted of an electropolished sample placed centrally between two electrodes (cathodes), 2 cm apart on each side. The 304 steel sample was also polished in a bath after long-term operation containing about 6% iron (304-EP6). After the electropolishing process, samples were subjected to multi-stage rinsing in distilled water. The samples, so prepared for further study, were then labeled as containing the type of steel (304 or 316), the surface treatment (AR—as received or EP—electropolished), and the content of Fe contamination in the electropolishing process bath (0 or 6 percent by mass).

### 2.3. AFM

An atomic force microscope was used for the observation of the passivation layer’s reconstruction process in the time domain. Such an approach was possible due to the unique abilities of AFM to utilize the scanning probe as both a tool for surface nanomodification and a measurement device enabling subnanometer-resolution surface 3D quantitative imaging [49,50]. Such unique ability, typical for correlational nanoscopy, enables multi-functional utilization of the setup in a specifically defined submicron area. The nanoscratching procedure, enabling oxidation layer recovery, was performed using a diamond-coated silicon probe DDESP (Bruker, Camarillo, CA, USA). The parameters of the used probe were as follows: material: 0.01 Ω·cm to 0.02 Ω·cm antimony (n) doped silicon; back coating: aluminum; tip coating: conductive diamond, r_tip_ = 150 nm, k = 42 N·m^−1^, f_res_ = 320 kHz. The tip’s diamond coating provided it with durability and wear resistance, which played an essential role in the experiment in terms of surface modification and long-term scanning. The surface imaging data analysis did not reveal any traces of scanning tip wear or contamination during the experiment. The experiment was performed in ambient conditions, while temperature and relative humidity were controlled and maintained at levels 25 °C ± 2 °C and 45% ± 4%.

**Figure 1:**
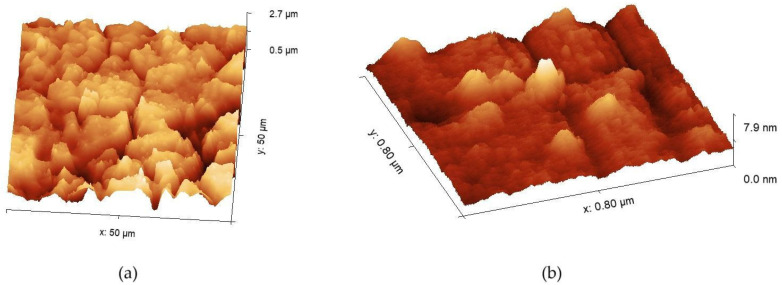
Examples of the 316-AR sample surface imaged by means of AFM: (**a**) showing the grainy structure of the material, (**b**) after the nanoscratching procedure.

The parameters of the nanoscratching procedure were selected based on previous experiments, where there had been various probe speeds and heights offset; hence, pressure forces were applied. It should be underlined that the individual processing parameters were related to a certain operation location, while specific grains required some slight approach modifications due to mechanical inhomogeneities of the material. One has to be aware that the force applied to the surface was the subject of a value limitation, while the probe with a certain spring constant must not apply the force, introducing the risk of cantilever damage and hence the destruction of the probe. These particular criteria lead to the different starting depths of the nanoscratches. One example of a 3D image of the sample surface showing the grains’ presence is shown in Figure 1a. A large area images were used to determine the spots where the experiment could be performed. The typical speed of the probe during the nanoscratching procedure was set to 0.5 μm/s, while the determined change to the probe’s height was set to 200 nm below the previously imaged surface level. The actual force used to modify the surface varied according to the observed area due to different grain orientations in the passivation layer. The above-mentioned procedure incorporated the fabrication of two parallel scratches, oriented vertically, while the fast scanning axis of the microscope was horizontal. An example of a 3D image of the sample surface showing grooves developed using the nanoscratching technique is shown in Figure 1b.

The successfully performed nanoscratching process was followed by the scanning procedure. In order to observe the long-term process of the passivation layer reconstruction, the optimal spot (presence of the surface plateau and lack of near debris) was chosen individually for each set of scratches. Once the location of the best measurement spot was determined, the repetitive profile’s acquisition process was launched by repetitive scanning of a single line (slow scanning axis disabled). The measurement was performed continuously, generating the set of files containing time domain surface evolution information. The obtained set of 2D maps provided a Y-axis time-related map of the cut depth changes. By measuring the depth of the fabricated trench at certain time steps, one was able to develop time-related graphs revealing the dynamics of the passivating layer reconstruction. The obtained data show the nanoscratches’ depth decrease progress, revealing the passivation layer reconstruction. The plateau level next to the sides of the nanoscratches, used as the reference level, does not change its height.

**Figure 2:**
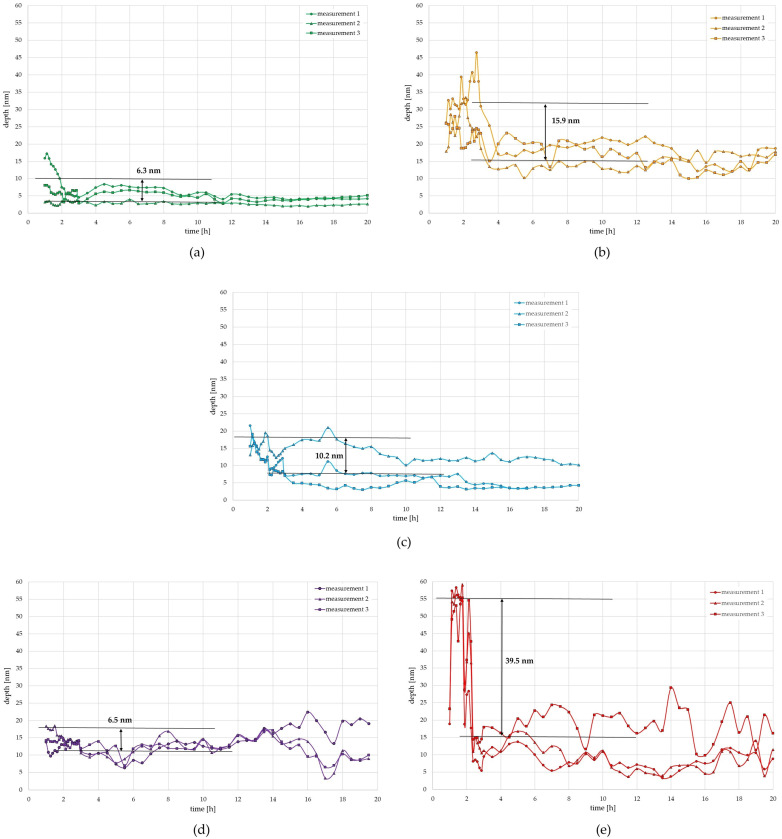
The graphs illustrate the passive layer reconstruction process in the time domain for the following samples: (**a**) 316-AR; (**b**) 316-EP0; (**c**) 304-AR; (**d**) 304-EP0; (**e**) 304-EP6.

Each procedure allowed the generation of approx. 70 files, covering a 24 h period (a single file contained 256 lines acquired within a 20 min time period). The data processing involved basic tilt correction and cut depth determination using profile analysis. In order to improve the statistical reliability of acquired data, 10 profiles were averaged. The results of the measurements are shown in Figure 2.

The above-mentioned procedure was performed at least three times in different locations, until three data sets were successfully acquired for a certain sample. Such an approach provided a more reliable statistical outcome, taking into account the local (single grain) properties of the sample.

### 2.4. XPS

XPS analyses were carried out according to the procedure and parameters as described in the authors’ previous work in the section Experimental procedures- XPS surface analyses [24]. The XPS analyses were performed using a PHI 5000 VersaProbe spectrometer (ULVAC-PHI, Hagisono, Chigasaki, Kanagawa, Japan) based on the procedures developed at the Institute of Physical Chemistry, Polish Academy of Sciences [51,52].

## 3. Results and Discussion

The constant advancement in SS technology, which is compelled by the growing demands for operational parameters of different SS alloys, is a key driver among researchers to better comprehend the complexities of the processes taking place on the material’s surface. It has been noted that variations in surface modification happen continuously. In this paper, we presented the application of the advanced AFM technique supplemented by the XPS technique. AFM was offered as a technique for monitoring the passivation layer reconstruction process on the surface of SS, before and after electropolishing. To achieve this, a method known as nanoscratching was employed, which involves using a diamond scanning probe to create scratches on a material’s surface, breaking the passivation layer’s continuity. The AFM application appears to be a very helpful instrument in the analysis of the impact of various conditions on these phenomena, since it makes it possible to observe the dynamics of the rebuilding of the passivation layer in a quantitative manner. AFM observations of the SS passive layer reconstruction rate after nanoscratching enabled the evaluation of changes in the depth of scratches with a subnanometer resolution (Figure 2). The depth provided in Table 1 was calculated as the difference between the initial depth of the nanoscratch and the value where no further change was observed.

Table 1 summarizes the AFM results and passive layer composition based on XPS depth profiles for the studied samples. Higher carbon percentages of 5.1% and 6.9% were observed for the as-received samples, for steel 316 and 304, respectively. The samples after the electropolishing process had passive layer carbon contents of about 1.4% or less. Similarities between the as-received samples and electropolished samples have also become apparent for the iron and chromium content. The as-received samples obtained higher amounts of iron in the passive layer along with lower chromium values than the electropolished samples, for which less iron and more chromium were noted. It can be seen that the Cr/Fe ratio values were similar, which were 0.82–0.86 for the as-received samples. The electropolishing process increased the chromium/iron ratio. The highest values of close to 1.9 were reached by 304 steel after the electropolishing process in a bath containing 0% contaminants. The other two samples, after the electropolishing process, achieved similar Cr/Fe ratio results of about 1.4. It should be noted that the test samples initially had a lower chromium content in 316 steel than in 304 steel. However, the addition of molybdenum in 316 steel significantly distinguishes it from 304 steel.

In the literature, various techniques have been used in relation to the determination of the dynamics of passive layer changes. Zhang et al. [53] used the TEM (Transmission Electron Microscopy) technique to image passive film growth on 316 stainless steel in 0.1 M NaOH solution. The researchers observed the presence of an ultra-thin film ~4 nm thick on the surface of stainless steel needles after polarization at −0.65 V for 7200 s. Furthermore, they determined that the Fe/Cr ratio increased with increasing applied potential. High-resolution TEM was also used by Tong et al. [54] for studies conducted for AISI 304 L austenitic stainless steel in a borate buffer solution. The authors reported that the thickness of the passive layer was 10–15 nm, 2–4 nm, and 10–18 nm for samples that were in contact with the solution for 1.5 h, 3 h, and 8 h, respectively. They described the results as consistent with those obtained by the transient corrosion current i_OCP_ and photocurrent i_ph_ during passive film growth.

Mujanović et al. [1] have shown that after mechanical depassivation in scratch tests on various stainless steels, repassivation at open circuit potential takes place within the first few tenths of a second after scratching. However, the final healing of the passive layer as well as reaching a steady passive state take much longer. According to our research, the reconstruction of the passive layer of samples 304 and 316 SS took 1–3 h. The passive layer growth rate is higher at the start of the study and decreases over time. Both the width and depth of the cut affect the process’s kinetics. Lv et al. [55] used both TEM imaging and EIS (electrochemical impedance spectroscopy) corrosion resistance testing techniques to study the surface of AISI 304 steel. TEM studies conducted for samples cold-rolled at different temperatures made it possible to determine that the sample cold-rolled at −120 °C had the most uniform microstructure. Moreover, EIS studies showed the highest phase degree for this sample, indicating that its passive film is more capacitive, hence a more condensed and intact film. The authors concluded that of the surfaces tested, this one (120 °C) had an equiaxed microstructure and displayed the best corrosion performance. The EIS technique has been employed to evaluate 316 L stainless steel passive film grown in acidic solution by Boissy et al. [56]. The authors used a developed special electrical equivalent circuit for testing at 20 °C, 60 °C, and 80 °C. At the given temperatures, the created circuit made it possible to calculate a diffusion coefficient of about 10^−14^ cm^2^/s, which was in line with the values presented in the literature.

Based on literature reports and our own research, it was found that in the case of 304 and 316 stainless steels, the passive layer can usually have a thickness of a few to several nm. However, as stated by [57], the 304 L stainless steel’s surface condition influences the semiconductor’s properties as well as the passive layer’s thickness. Similarly to [53], we observed that the compositions of the passive film were mainly Cr and Fe oxides, based on evaluation of the XPS results.

**Figure 3:**
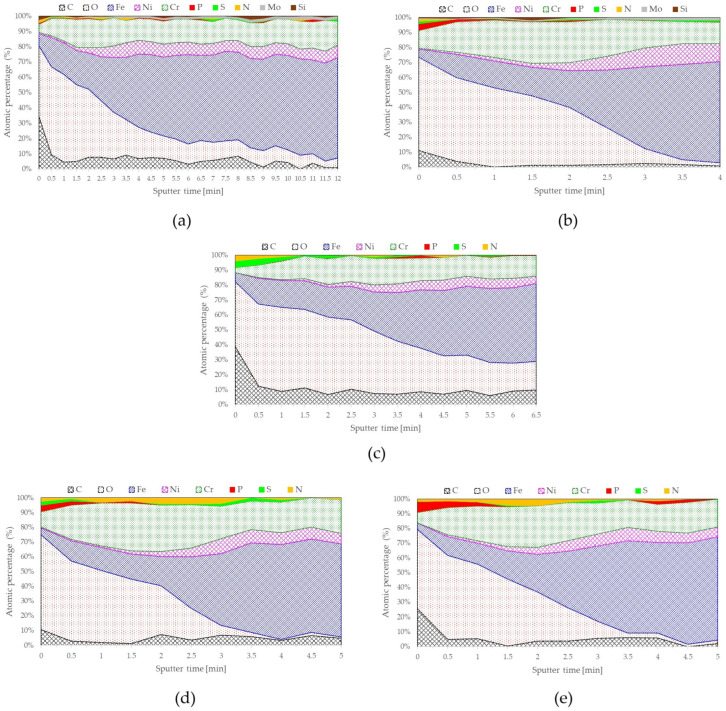
Depth profiles of the surface of samples: (**a**) 316-AR; (**b**) 316-EP0; (**c**) 304-AR; (**d**) 304-EP0; (**e**) 304-EP6.

XPS tests were used to analyze the chemical composition of the samples’ surfaces. The tests were carried out for samples made of two types of steel (304 and 316), with both as-received surfaces and after electropolishing in baths with various amounts of contaminants—0% and (for 304 SS) 6% Fe by mass. The chemical composition (at %) of the surface is shown in the depth profiles for the analyzed samples of 316 steel (Figure 3a,b) and 304 steel (Figure 3c–e). The contents of individual elements in the passive layer are shown as percentages. After 2–3 min sputtering the surface with Ar^+^ ions, a decrease in chromium content can be observed, with a simultaneous increase in nickel content. This is clearly visible for the samples after the electropolishing process. For all samples tested, the iron content is higher than the chromium content in the bulk. The passive layer composition of both 304 and 316 steels is characterized by a substantially greater Cr/Fe content for the samples after the electropolishing procedure as compared to the value before electropolishing. Furthermore, it was found that the electropolishing process could still be carried out in polluted electrolytes with up to 6% mass iron ions in the electrolyte composition. Despite the significant contamination of the electropolishing bath used, the surface obtained after the process was even characterized by an enhanced composition of the passive layer compared to the surface finish 2B for the as-received samples.

**Figure 4:**
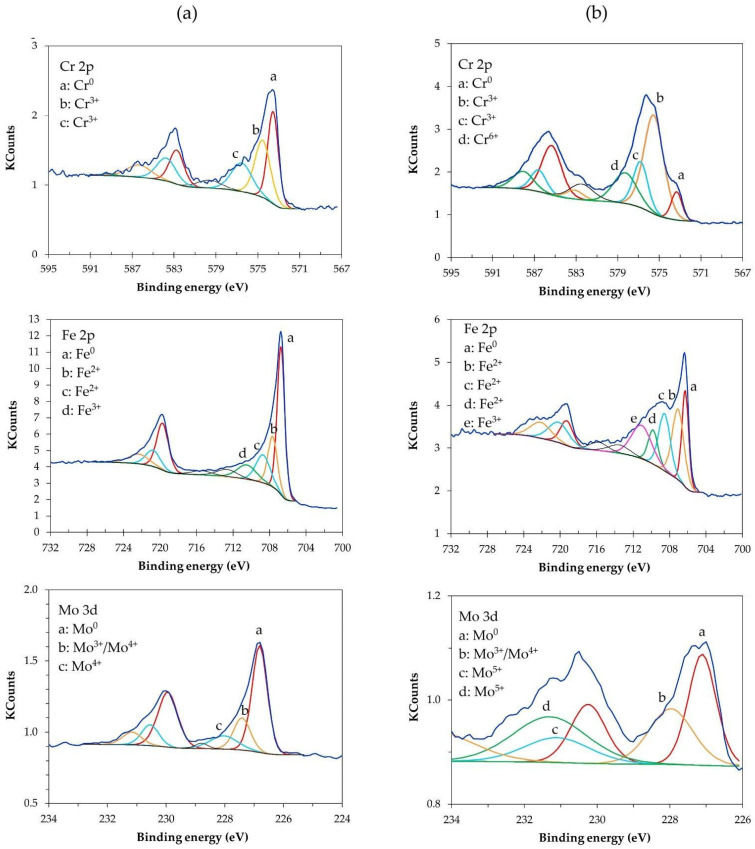
XPS Cr2p, Fe2p, Mo3d deconvolutions of the analyzed samples: (**a**) as-received 316 sample after sputtering for 2.0 min with Ar^+^, (**b**) electropolished 316 sample after sputtering for 1.5 min Ar^+^.

Cr 2p3/2, Fe 2p3/2, and Mo 3d5/2 spectra after sputtering with Ar^+^ are presented for samples 316-AR and 316-EP0 (Figure 4). Deconvolutions for samples made of 304 steel and subjected to electropolishing were published by the authors of a previous article [26].

Deconvolutions corresponding to Fe^2+^ oxides are visible for the as-received sample at 707.6 eV and 708.8 eV and for the electropolished sample at 707.0, 708.5, and 709.8 eV (FeO). Peaks corresponding to Fe^3+^ (Fe_2_O_3_) oxides are present for the as-received sample at 710.6 eV and for the 316-EP0 sample at 711.1 eV. The results obtained in the authors’ previous studies for electropolished 304 steel samples indicated the presence of Fe^2+^ oxides at 709.1–709.2 eV and Fe^3+^ oxides at 710.6–711.3 eV [26]. A peak, indicating iron, in metallic form was observed at 706.8 eV and 706.3 eV for the as-received and electropolished samples, respectively. Other researchers have indicated the presence of XPS peaks of metallic iron at 706.6 eV [58]; Fe^2+^ (FeO) between 709.0, 709.6, and 709.7 [59,60]; and Fe^3+^ oxides (Fe_2_O_3_) between 710.5 and 711.5 eV [60].

Chromium is present in metallic form at 573.5 and 573.1 for the as-received and electropolished samples, respectively. In addition, there are peaks corresponding to Cr^3+^ visible for sample 316-AR at 574.3 and 576.5 eV and at 575.5 eV, 576.7 eV for the electropolished sample, as well as for Cr^6+^ (CrO_3_) at 578.4 eV, presented for sample 316-EP. Previous studies by the authors have suggested the occurrence of chromium Cr^3+^ oxides on the surface of 304 steel samples in a range of 576.0–576.7 and Cr^6+^ oxides in a range of 578.6–578.9 eV [26]. The values obtained for 316 steel samples are very similar to the previously obtained results. Lai et al. [58] and Yi et al. [61] noted the presence of chromium(III) oxides at 576.6 eV. Other reports suggest the presence of Cr^3+^ oxides (Cr_2_O_3_) at 576.0–576.8 eV [62,63] and Cr^6+^ (CrO_3_) at 578–578.3 eV [64].

Signals associated with molybdenum appear in a range of about 227 to 229 eV. For both the as-received sample and that after the electropolishing process, the signal associated with the metallic form of Mo is visible at 227.5 eV and 227.3 eV, respectively. For both samples, a second peak is observed that is visible at 228.1 eV, and it is not clear which form it may indicate, but it could be associated with the presence of Mo^3+^ or Mo^4+^. The presence of Mo^4+^ in the form of MoO_2_ was noted in the as-received sample at 228.7 eV. For an after-electropolishing sample, two peaks are present at 231,1 eV and 231,3 eV, which can be associated with the Mo^5+^ form. For the electropolished sample, a peak indicating the presence of Mo^4+^ oxide (MoO_2_) is seen at 231.5 eV. Other authors conducting tests with 316 steel have noted the presence of XPS peaks associated with the metallic form of molybdenum at 227.0–227.1 eV [65] and 227.8 eV [66]. Molybdenum oxide (MoO_2_) peaks were present at 228.6–229.1 eV in a study by Bortolan et al. [65], while Mo^5+^ peaks were located at 231 eV according to data in a study by Wang et al. [66].

## 4. Conclusions

Based on a reconstruction of the passive layer of AISI for as-received samples from 304 and 316 steel and after electropolishing of these steels, samples of 304 and 316 steel after scratching needed between 1 and 3 h to reconstruct the passive layer. During this time, there was a stage of reconstructing the thickness of the passive layer at a level of a few to several nanometers. AFM measurements in the following hours up to the 20th hour did not indicate any further significant changes in the passive layer’s thickness. The recovery of the passive layer was markedly different for the 304 steel sample, which had previously been electropolished in an extremely heavily contaminated bath to an iron ion content of 6 wt.%. Between 1 and 2 h after scratching, a thickness of 40 nm was needed. A further several hours confirmed, as with the other samples, that there was no further change in the layer’s thickness. The study indicates that irrespective of the level of contamination of the process bath, AISI 304 and 316 steels do not lose their ability to rebuild the passive layer after scratching, even though the level of contamination may affect the rate of rebuilding the passive film. In addition, resistance to the nanoscratching procedure varied for the tested samples, as one could see differences in the depth’s starting values. In particular, one might notice that the electropolishing process has increased the submicron homogeneity, with the AFM experiments revealing a much more significant deviation of the initial nanoscratching result, while the processed samples revealed a better repeatability of the outcome. Also, the electropolishing process seems capable of inflicting damage with relative ease on the passivation layer through nanoscale scratching, while the AR samples did not reveal so deep a cut.

Based on our own XPS studies and the relevant literature, the passive film on both AISI 304 and 316 steels is usually a few to several nm thick. The passive layer of austenitic steels owes its resistance and ability to regenerate to the content of chromium oxides and hydroxides; in addition, AISI 316 contains molybdenum oxides in the passive layer formed. XPS studies have shown that the composition of the passive film for both 304 and 316 steels is characterized by a significantly higher Cr/Fe content of 1.41–1.88 for samples after electropolishing compared to values of 0.82–0.86 for as-received samples. Even extremely long electrolyte operation at up to a value of 6 wt.% of iron ions in the electrolyte composition still enables the electropolishing process, resulting in an improved passive layer composition compared to the surface finish 2B for the as-received samples.

## Figures and Tables

**Table 1:** Summary of AFM results and XPS passive layer composition based on depth profiles.

Sample	AFM	XPS	XPS	Cr/Fe Ratio	C	O	Fe	Ni	Cr	P	S	N	Mo	Si
	Reconstruction	Sputter Time	Etch Depth											
	[nm]	[min]	[nm]	[-]	[at%]	[at%]	[at%]	[at%]	[at%]	[at%]	[at%]	[at%]	[at%]	[at%]
316-AR	6.3	1.5	2.06	0.82	5.1	50.0	22.3	2.1	18.3	0.5	0.0	0.3	0.4	0.9
		2	2.74	0.80	7.7	44.6	23.7	3.4	19.0	0.4	0.0	0.5	0.7	0.0
316-EP0	15.9	1.5	2.06	1.45	1.4	46.4	19.2	2.4	27.9	0.2	0.1	0.0	0.5	1.9
		2	2.74	1.12	1.4	38.7	24.5	5.3	27.4	0.9	1.1	0.0	0.6	0.2
304-AR	10.2	1.5	2.06	0.79	11.4	52.5	19.2	1.2	15.2	0.0	0.5	0.1	-	-
		2	2.74	0.86	6.9	51.7	20.1	1.6	17.4	0.2	2.0	0.1	-	-
304-EP0	5.6	1.5	2.06	1.88	1.3	43.5	17.2	2.1	32.4	1.1	0.0	2.4	-	-
		2	2.74	1.58	7.4	32.9	20.0	3.5	31.5	0.0	0.5	4.3	-	-
304-EP6	39.5	1.5	2.06	1.41	0.4	45.3	19.2	2.9	27.0	0.2	0.8	4.3	-	-
		2	2.74	1.10	3.8	33.1	25.7	4.6	28.2	0.0	0.0	4.7	-	-

## Data Availability

The original contributions presented in this study are included in the article. Further inquiries can be directed to the corresponding author.
